# New English Anesthetic Legislation

**Published:** 1909-07-15

**Authors:** Edward C. Kirk


### NEW ENGLISH ANESTHETIC LEGISLATION.

BY EDWARD C. KIRK, D.D.S., S.C.D.

From the Proceedings of the Royal Society of Medicine, Section of Anesthetics, meetings of January 20 and February 5, 1909, we gather the information that the General Medical Council of Great Britain has, in general terms, given its approval to a proposed parliamentary enactment entitled "General Anesthetics Act, 1908," which provides that no "person other than a legally qualified medical practitioner" shall be permitted to administer or cause to be administered to any other person, by inhalation or otherwise, any drug or substance, whether solid, liquid, vaporous, or gaseous, and whether pure or mixed with any other drug or substance, with the object of producing a state of unconsciousness during any medical or surgical operation, act, or procedure, or during childbirth," under a penalty, after conviction, of ten pounds for the first offense, and in case of a subsequent conviction a penalty of twenty pounds.

Section 2 of the proposed act provides that all examining bodies recognized by the General Medical Council of Education shall require of all candidates for qualification evidence of thorough instruction in anesthetics, both theoretical and practical, and that they shall have personally administered anesthetics under the supervision and to the complete satisfaction of their respective teachers.

Section 3 exempts all dentists registered under the act of 1878 and before the passage of the present act to the extent that they may use anesthetics as required during any dental act or procedure. That is to say Section 3 of the proposed act permits the use of anesthetics by dentists who were registered after the act of 1878 was passed, but excludes from the use of anesthetics all dentists who shall register after the passing of the proposed General Anesthetics

Act here under consideration; it regards the privilege of administering anesthetics enjoyed by dentists under the act of 1878 as a vested right, but the new bill proposes to abrogate that right for all dentists who register after its passage.

We do not know who is responsible for the drafting of this extraordinary measure, so that what we may say of it in a critical way is quite impersonal in its application, but taken as a whole it strikes us as an amazing example of attempted legislative stupidity. Shades of Horace Wells, Morton, and Simpson—to say nothing of Sir Humphry Davy! Anesthesia a dental discovery—the greatest boon ever bestowed upon humanity for the relief of agony and suffering, given to the world by dentists—and now a few superserviceable medical enthusiasts of monopolistic tendencies would arrogate to themselves the exclusive right to dispense its blessings.

But the supporters of the scheme contend that fatalities occur from the administration of anesthetics by the unskillful. Dr. Hewitt reports* no less than *six* "*deaths* from nitrous oxid in the last *twenty-one years* in the hands of persons with no medical qualification." Unfortunately the same speaker was not called upon to report the number of deaths in Great Britain from chloroform administrations given by "persons" with a "medical qualification" during the same period. It is regrettable that these figures were not also demanded; the comparison would have been interesting and instructive. To stand complacently watching the increasing record of chloroform fatalities piling up with grim regularity year by year, and at the same time burst out in a spirit of pious regard for the public weal by attempting to prevent the extraction of teeth under nitrous oxid anesthesia unless the patient is able to pay the fee to a qualified medical man for dispensing it, is a picture of consistency that would be humorous if it were not pathetic. It is true that it is difficult to obtain reliable statistics cov-

* *Vide* Section of Anesthetics, Proceedings Royal Society of Medicine, vol. ii, No. 4.

ering the administration of anesthetics, but it is certainly well known that deaths from chloroform outnumber both absolutely and relatively those caused by any or all of the anesthetics in common use. One death to four thousand administrations is the lowest rate which careful observers can claim for chloroform, whereas estimates give one death in a quarter of a million administrations as being well within the record as to nitrous oxid anesthesia. Besides which, chloroform is as a rule administered by qualified medical men, whereas nitrous oxid is administered mainly by dentists, some qualified, many not qualified at all except by the courage of their ignorance. The death-rate from nitrous oxid is less than the death-rate from the operation of British railways, and probably more people are killed by street accidents in London in one day than in a lifetime of nitrous oxid administrations. Or, more to the point, it is probably true that more people die from the operation of tooth-extraction and the conditions which necessitate the operation than die from the inhalation of nitrous oxid. Considering the many cases of unrecognized heart trouble, and the avoidance of shock which nitrous oxid secures, it is the opinion of competent students of the question that it is safer, all things considered, to extract a tooth under nitrous oxid anesthesia than to perform the operation without it;—therefore, why not draft a bill to prevent dentists from extracting teeth?

It is inconceivable that this proposed act will receive the serious consideration of the British Parliament, or that it would receive serious consideration by any body of men who were capable of seeing beyond the limits of a narrow specialism. The bill contains a clause providing for a more thorough training by all who use anesthetic agents for the purpose of "producing unconsciousness." This feature of the proposed act is admirable as far as it goes, but in principle it is like requiring a poison label to be placed upon the corrosive sublimate bottle and a few others, like strychnia and arsenic, in a chemist's shop, and letting the others speak for themselves, the natural inference by the uninformed being that the contents of the bottles without the poison label are harmless. The analogy arises from the fact that the proposed act applies only to drugs used for producing unconsciousness by inhalation; but what is to be done about cocain and its salts? Is the dentist or the otherwise medically unqalified person to be permitted to use cocain and allied analgesics without let or hindrance? Is not the death-rate from cocain injections as great or greater than from nitrous oxid? Are not the injuries and surgical disorders following the ignorant and empirical use of local anesthetic drugs by injection as pertinent conditions for legislative regulation as nitrous oxid anesthesia?

About once in so often there is an outburst of medical righteousness which manifests itself in an attempt to regulate things by law so that the public will be driven more definitely under medical control under the assumption that the dear public cannot be very safely trusted to look after its vital affairs. Medical legislation has gone so far that the reproduction, vital maintenance, and ultimate departure of the human race, in civilized communities at least, is in its main features under medical supervision. As a general proposition it may be conceded that some supervision of what is generally grouped under the term "the public health" is needed, but on the other hand the public can, to a limited extent at least, be trusted to take care of its own health interests, and as on the whole it can be very clearly shown that the public has not suffered very great damage by the administration of nitrous oxid even by "persons without a medical qualification," and as its casualities resulting from the ministration of persons with a medical qualification are known to be infinitely more numerous, we are of the opinion that no serious-minded or practical body of legislators will be found either in England or elsewhere who will feel that circumstances will warrant them in passing an act depriving the public of the comforts and benefits of nitrous oxid anesthesia in dentistry excepting at the behest and under the sanction of the medical practitioner.

We have drawn attention to this proposed legislation because of the medical attitude of mind which it represents, an attitude which presupposes that a medical qualification is a guarantee of safety in all that concerns public health, and by inference that the dentist is not qualified to take care of those features of the public health that naturally fall within his province. The general public is better informed as to the facts in the case, and not being influenced by professional prejudice or professional interest in such matters can be trusted to form its own conclusions, and through its representatives in legislative bodies to defeat any such narrow-minded attempts at class legislation as they arise.—*Cosmos.*

